# Anatomic Posterolateral Corner Reconstruction With Single Tibialis Allograft and Suspensory Tibial Fixation

**DOI:** 10.1016/j.eats.2024.103265

**Published:** 2024-09-27

**Authors:** Brandon Cabarcas, Raahil Patel, Raegan Mahler, Neil Kumar, Drew Warnick

**Affiliations:** a Department of Orthopaedic Surgery, University of South Florida, Tampa, Florida, U.S.A.; b Children’s Orthopaedic and Scoliosis Surgery Associates, St. Petersburg, Florida, U.S.A.

## Abstract

Injuries to the posterolateral corner (PLC) of the knee are difficult challenges for orthopaedic surgeons to manage. The original LaPrade anatomic reconstruction technique using 2 allografts from a split Achilles tendon has become a well-recognized reconstructive approach for addressing injury to the PLC. In this article, we present our modified technique of anatomic PLC reconstruction using a single tibialis allograft and suspensory tibial fixation. We describe the tunnel position, graft passage, and fixation strategies to achieve anatomic reconstruction of the 3 main stabilizers of the PLC (fibular collateral ligament, popliteus tendon, and popliteofibular ligament) while using a single allograft tendon. Suspensory fixation on the tibia is used to allow for individual tensioning of each limb of the reconstruction in an optimal knee position. We review the associated advantages, challenges, and technical pearls and pitfalls associated with our technique. We consider this modified approach a viable alternative to achieve a stable PLC reconstruction while still abiding by the established core anatomic reconstructive principles.

The posterolateral corner (PLC) of the knee has gained attention in the orthopaedic literature. The PLC structures, predominantly the lateral collateral ligament, popliteofibular ligament, and popliteus tendon, provide knee stabilization by resisting varus stress and external rotation.^1^^,^^2^ PLC injuries are challenging to diagnose, often presenting with anterior or posterior cruciate ligament injuries and rarely occurring in isolation (only 13%-28%).^3^, ^4^, ^5^, ^6^ Surgical reconstruction typically yields better outcomes than primary repair, although repair may be suitable for large bony avulsions.^2^^,^^7^ Indications for PLC reconstruction include acute or chronic grade III injuries (disruption of all PLC structures and varus laxity) and avulsions for which repair is not possible or in which the tissue is inadequate.^8^ Various anatomic and nonanatomic techniques for PLC reconstruction exist.^9^ The anatomic technique of LaPrade et al.^3^ using split Achilles tendon allograft is well recognized for addressing PLC injury. Modifications to improve cost and efficiency include using single hamstring autograft or multiple hamstring autografts, alternative allografts, and different surgical exposures.^10^, ^11^, ^12^, ^13^ We present our technique for anatomic PLC reconstruction using a single tibialis anterior or posterior allograft with suspensory tibial fixation.

## Surgical Technique

### Patient Positioning

**Fig 1:**
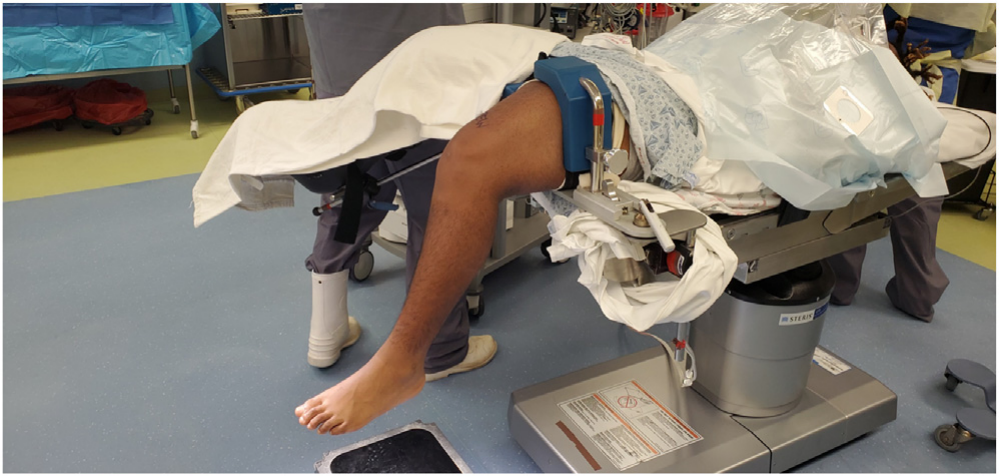
The patient is placed in the supine position with the operative extremity in a leg holder and the nonoperative extremity in a well-padded stirrup. The lower end of the table has been removed to allow the leg to hang freely.

The patient is placed supine on the operating table, and general anesthesia is administered. An examination under anesthesia and/or stress radiography is performed to confirm the clinical diagnosis. A well-padded pneumatic tourniquet is placed on the upper thigh, and the operative extremity is placed in a leg holder (Fig 1). The nonoperative limb is padded and placed into a stirrup in an abducted position to allow for intraoperative anteroposterior and lateral radiographs.

### Graft Selection and Preparation

**Fig 2:**
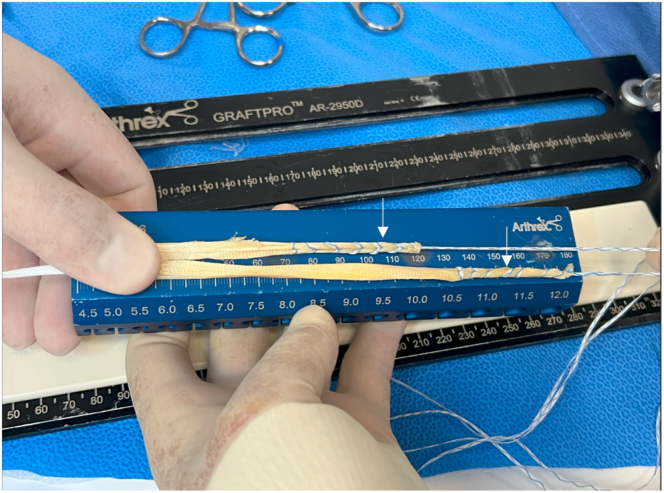
Single tibialis anterior allograft with FiberLoop whipstitches placed on both ends of graft (arrows) and TightRope in graft split.

**Fig 3:**
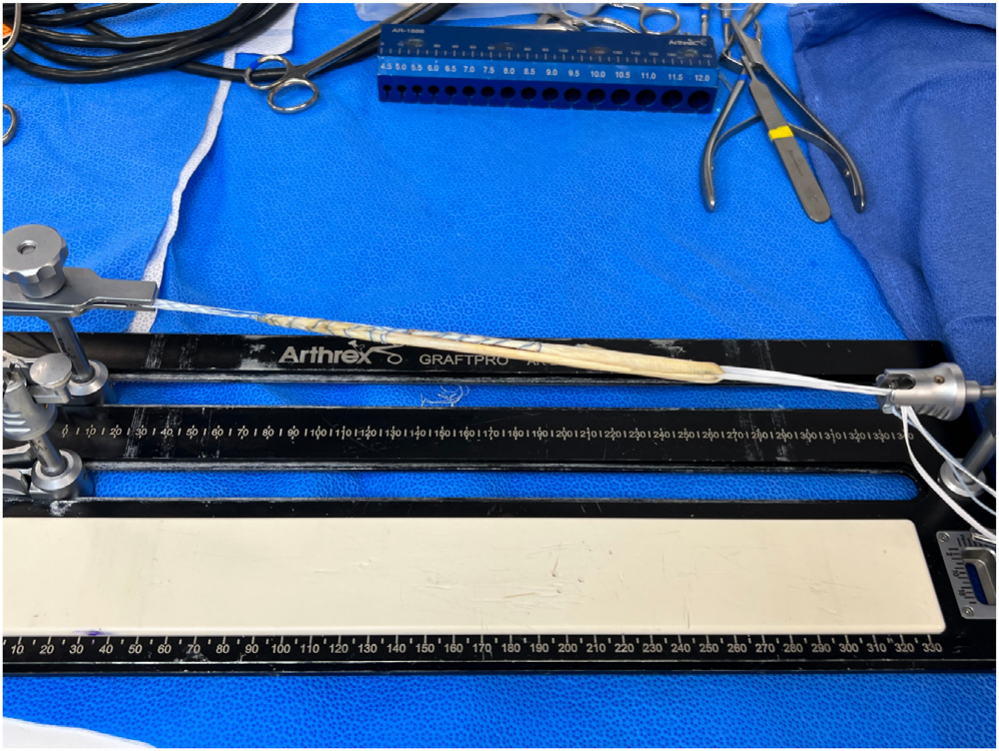
Single tibialis anterior allograft prepared with 2 FiberLoop whipstitches left) and TightRope button (right) secured to graft split. The graft is tensioned on the GraftPro preparation system appropriately before proceeding with the case.

For this technique, a single tibialis anterior or tibialis posterior allograft is chosen (Video 1). A graft length of 28 cm is greater is ensured prior to starting the procedure. The graft is prepared on the GraftPro preparation system (Arthrex, Naples, FL). A FiberLoop (Arthrex) whipstitch is placed on both ends of the graft using approximately 6 to 8 passes, with the last throw locking the whipstitch. The redundant graft tissue is trimmed from the edges. The graft is passed through the loop of the ACL TightRope (Arthrex) suture card to produce 2 limbs with minimum lengths of 12 cm and 16 cm on each side (Fig 2). Each limb should fit through a 7-mm tunnel. Two independent simple FiberWire (Arthrex) stitches are placed in the split of the graft to prevent sliding. The graft is then tensioned on the GraftPro preparation system appropriately before proceeding with the case (Fig 3).

### PLC Reconstruction

**Fig 4:**
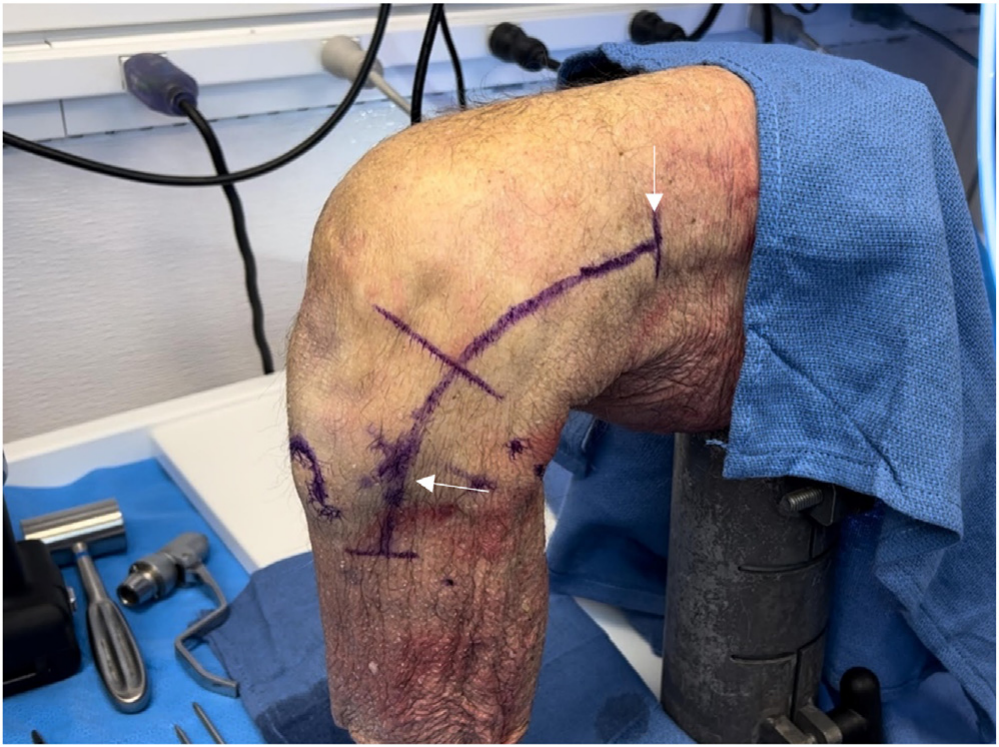
Lateral view of left knee in flexed position. A laterally based incision is made, beginning proximally along the iliotibial band (vertical arrow) and extending distally toward the Gerdy tubercle (horizontal arrow), between the fibular head and tibial tubercle.

**Fig 5:**
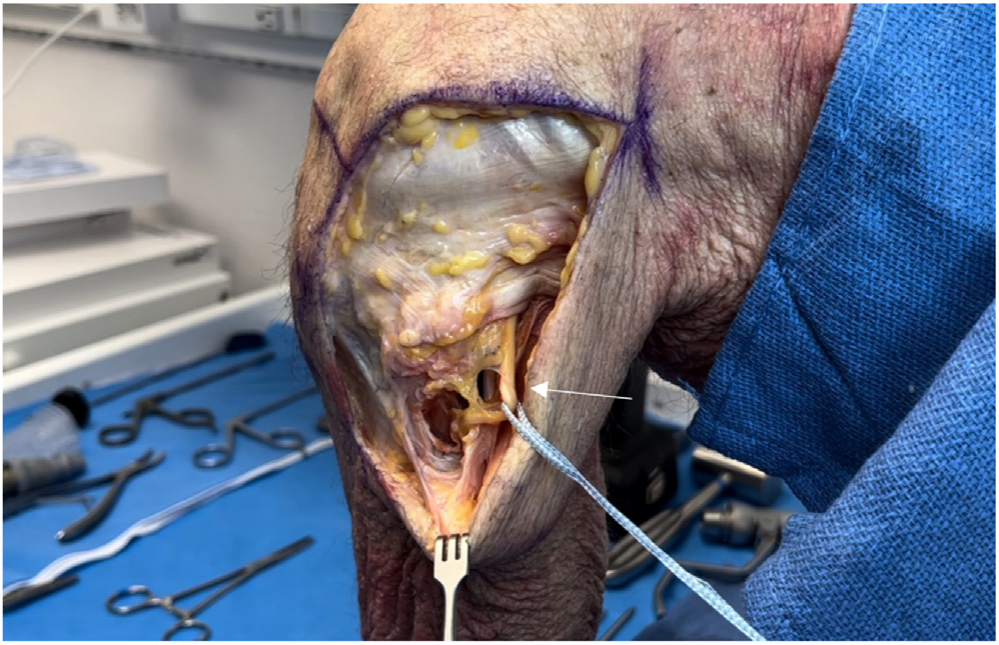
Lateral view of left knee in flexed position. The location of the common peroneal nerve is identified by palpation just distal to the fibular head (arrow). The nerve is carefully dissected proximally posterior to the biceps femoris tendon and subsequently tagged (with blue suture) to protect it throughout the procedure.

A laterally based incision beginning proximally along the iliotibial band (ITB) and extending distally toward the Gerdy tubercle is used (Fig 4). Once dissection has been carried down to the fibular head, the location of the common peroneal nerve is identified by palpation. The nerve is carefully dissected and followed proximally posterior to the biceps femoris tendon and is subsequently tagged to protect it throughout the procedure (Fig 5).

**Fig 6:**
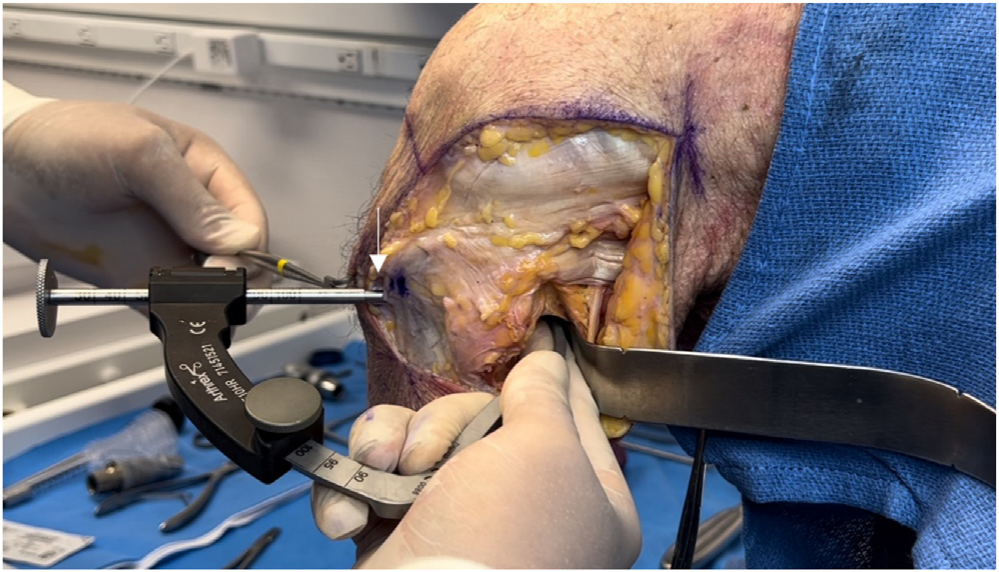
Lateral view of left knee in flexed position. The Arthrex drill guide for the tibial tunnel (arrow) is placed halfway between the Gerdy tubercle and the tibial tubercle on the anterolateral tibia, aiming toward the posterior popliteal tibial sulcus on the posterior surface of the tibia. A curved retractor is placed posteriorly to protect the neurovascular bundle.

**Fig 7:**
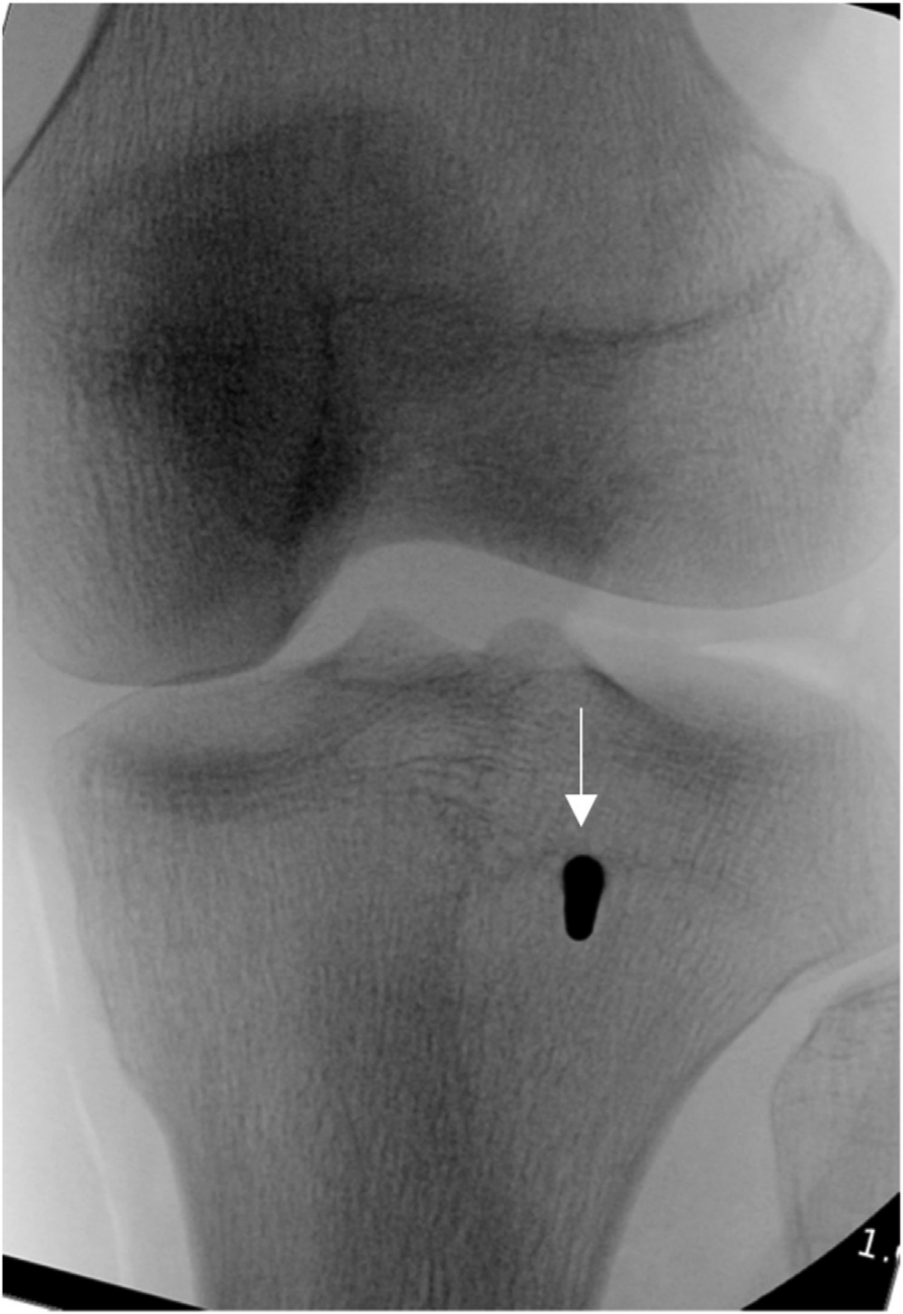
Anteroposterior fluoroscopic image taken down 3.5-mm-long drill-bit (Arthrex) trajectory used to drill into tibia from anterior to posterior (arrow) in left knee. The tip of the drill bit can be palpated posteriorly at this point, and its correct location verified anatomically.

The interval between the soleus and the lateral head of the gastrocnemius tendon is developed. The soleus muscle is bluntly elevated off the posteromedial aspect of the fibula toward the posterior surface of the tibia. The posterior tibial popliteal sulcus is identified by palpation approximately 10 mm distal to the margin of the articular cartilage.^1^ Next, sharp dissection is carried down the tibia between the Gerdy tubercle and the anterolateral tibia, and the fascia is lifted subperiosteally off the tibia for Arthrex drill guide placement. A curved retractor is placed posteriorly to protect the neurovascular bundle, and a drill guide for the tibial tunnel is placed, aiming toward the posterior popliteal tibial sulcus on the posterior tibia (Fig 6). A 3.5-mm drill bit (Arthrex) is directed from anterior to posterior and checked by palpation and radiographically (Fig 7). The FlipCutter reamer blade (Arthrex) is replaced and deployed to a diameter of 10 mm, and the tibial socket is drilled in a retrograde manner to a maximum length of 20 mm. A curved rasp can be used to clear the socket of any remaining bone or soft tissue to facilitate graft passage. A FiberStick (Arthrex) is used to pass the looped end through the tibial socket to create a passing luggage-tag stitch.

**Fig 8:**
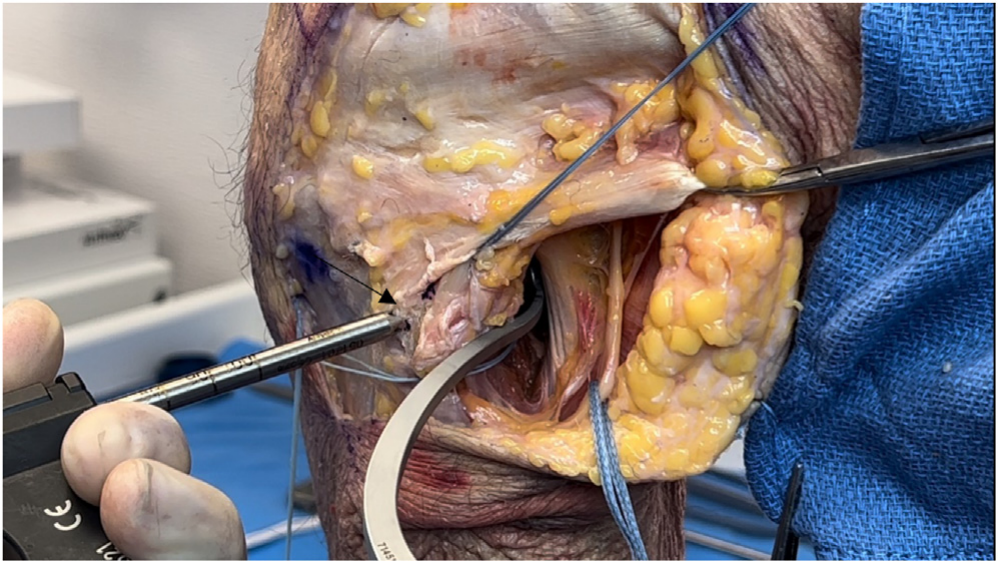
Lateral view of left knee in flexed position. After sharp elevation of the biceps femoris bursa and anterior aspect of its tendinous insertion, the distal attachment site of the fibular collateral ligament is identified on the fibular head. The Arthrex fibular drill guide is placed on the fibular collateral ligament attachment site on the lateral aspect of the fibular head (arrow), aiming toward the insertion site of the popliteofibular ligament on the posteromedial fibular styloid.

**Fig 9:**
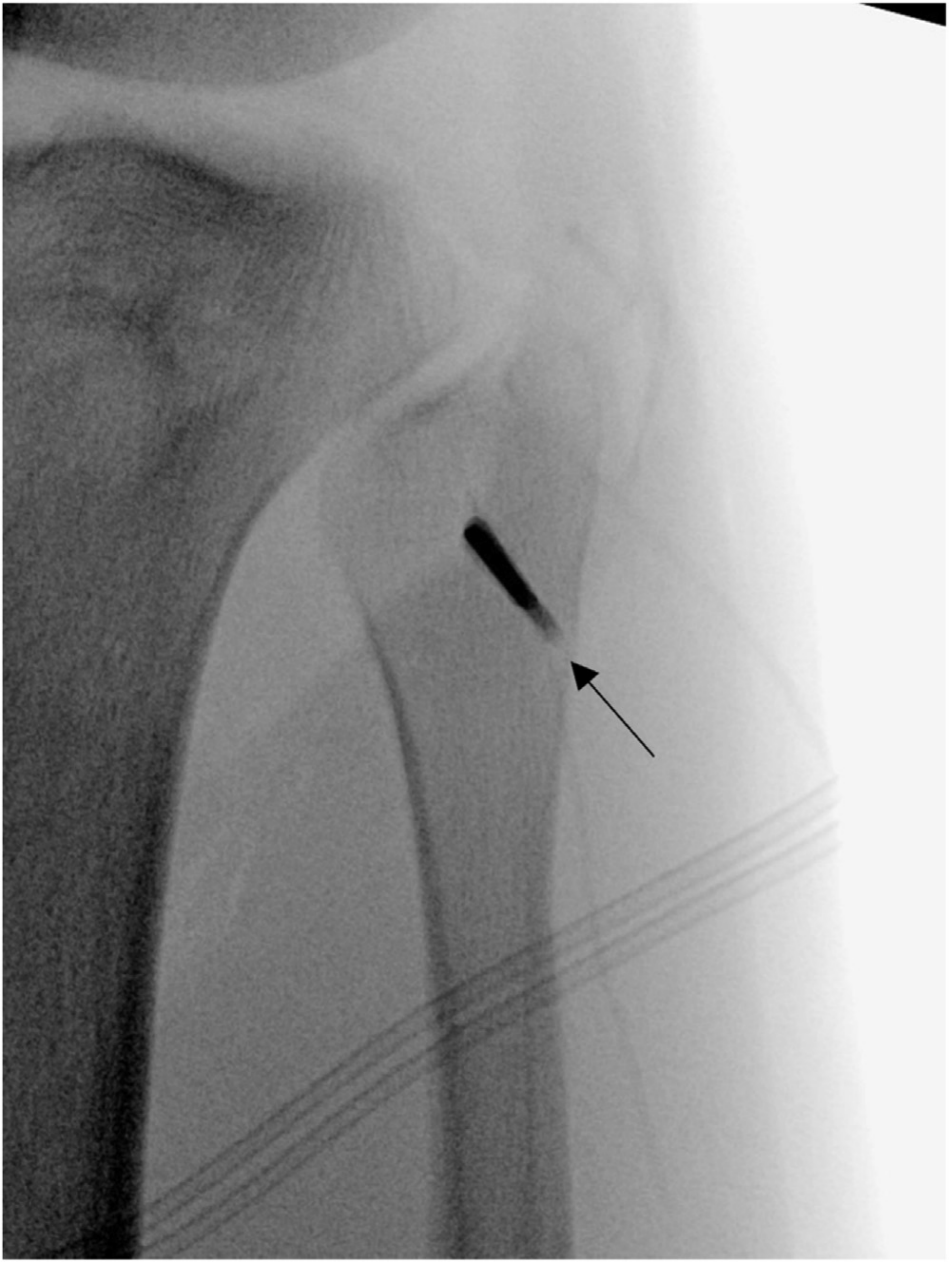
Anteroposterior fluoroscopic image taken down drill-bit trajectory in left fibula (arrow) to verify correct location and to avoid drilling out of fibular head and causing iatrogenic fracture.

After the fibular collateral ligament (FCL) is identified, a tagging stitch is placed in the FCL or remnant tissue. The anterior portion of the biceps femoris insertion on the fibular head is elevated to identify the distal attachment site of the FCL on the fibular head. The Arthrex fibular drill guide is placed on the distal attachment site of the FCL on the anterolateral aspect of the fibular head, aiming toward the insertion site of the popliteofibular ligament on the posteromedial fibular styloid (Fig 8). A 3.5-mm guide pin (Arthrex) is inserted, palpated on the posteromedial aspect of the fibula, and finally, confirmed using fluoroscopy. An anteroposterior image is taken down the drill-bit trajectory to verify the correct location radiographically and avoid drilling out of the fibular head (Fig 9). The guide pin is then over-reamed with a 7-mm cannulated reamer while the posterior neurovascular structures are protected with a curved retractor. This retractor is used to clean out the tunnel. A FiberStick is again used to pass the looped end through the fibular tunnel to create a passing luggage-tag stitch.

**Fig 10:**
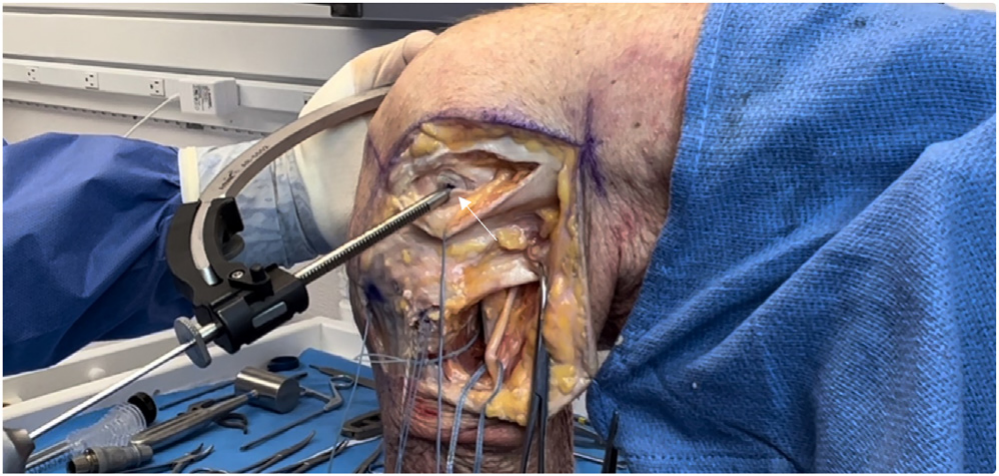
Lateral view of left knee in flexed position. The fibular collateral ligament attachment site just proximal and posterior to the lateral epicondyle on the femur (arrow) is identified after sharp dissection through the iliotibial band. A guide pin with a suture eyelet is drilled using the Arthrex drill guide, exiting the distal femur proximal and anterior to the adductor tubercle.

**Fig 11:**
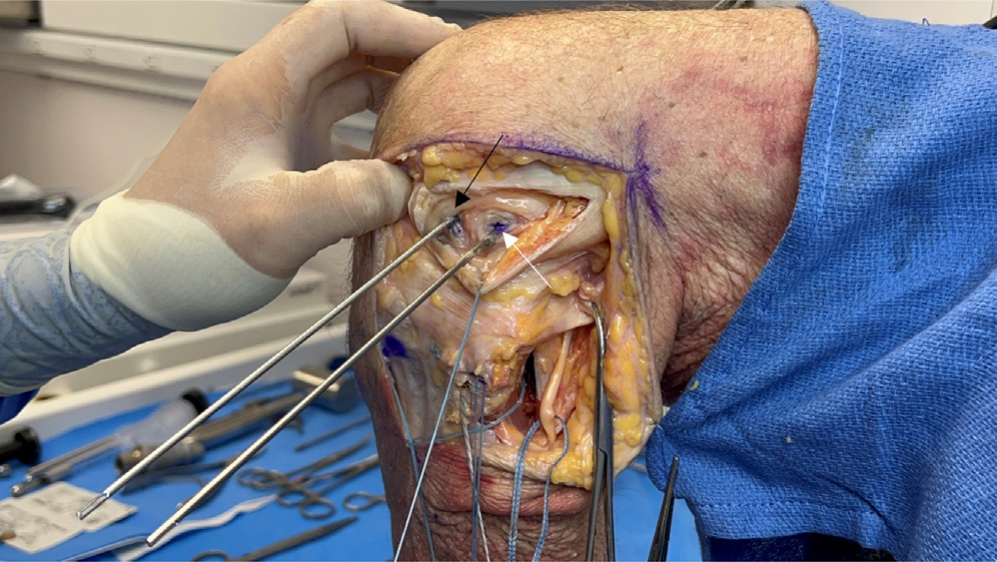
Lateral view of left knee in flexed position. After creating an arthrotomy approximately 2 cm anterior to the first guide pin (white arrow), a second guide pin is drilled through the distal femur using the Parallel Pin Guide (Arthrex) at the anatomic location of the popliteal insertion on the femur (black arrow) (approximately 18.5 mm distal and anterior to the fibular collateral ligament).

**Fig 12:**
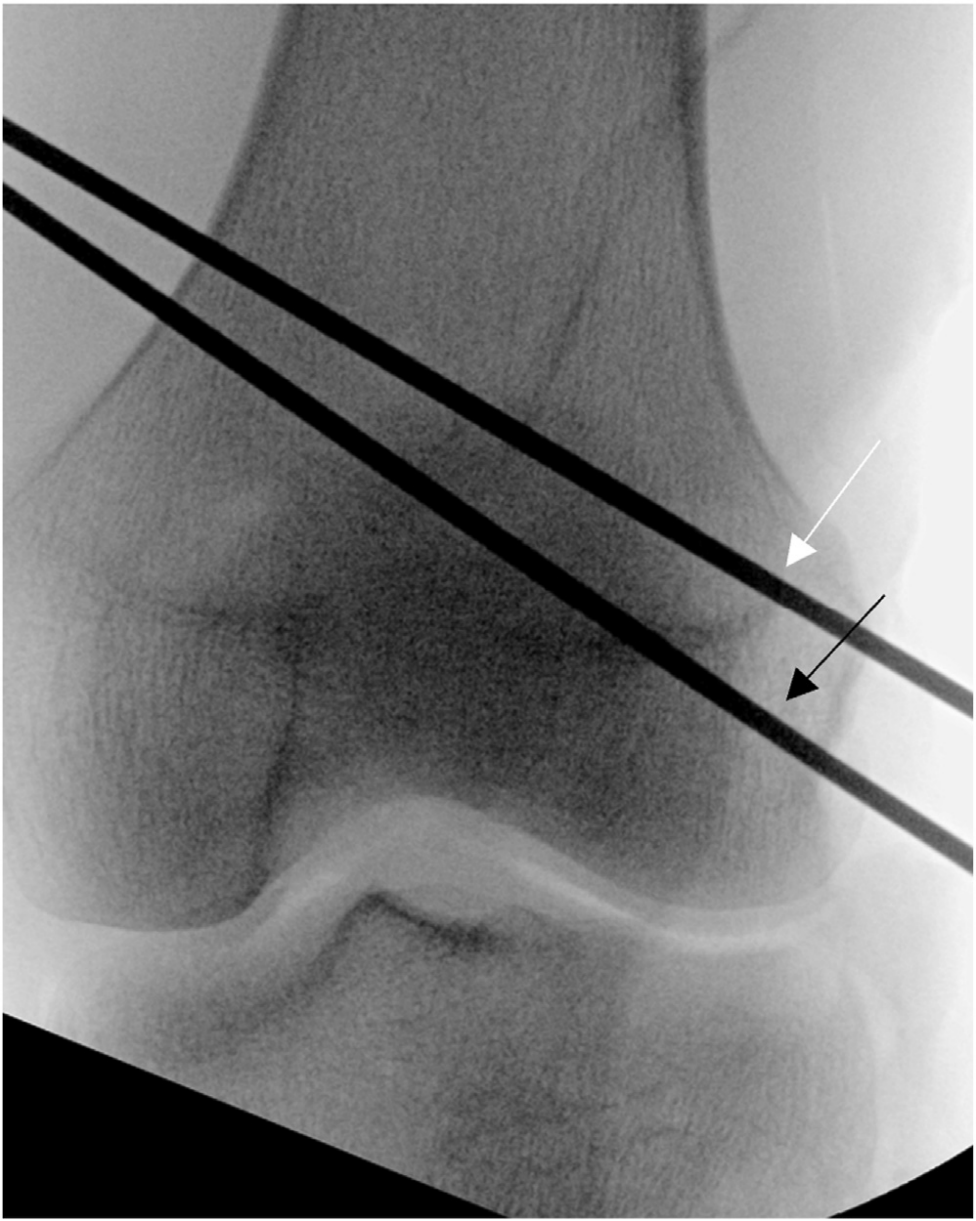
Anteroposterior fluoroscopic image of left knee with guide pins for fibular collateral ligament (white arrow) and popliteus (black arrow) insertions on femur drilled exiting distal femur proximal and anterior to adductor tubercle. This trajectory helps avoid any potential convergence with anterior and/or posterior cruciate ligament tunnels in the case of a multiligament reconstruction.

**Fig 13:**
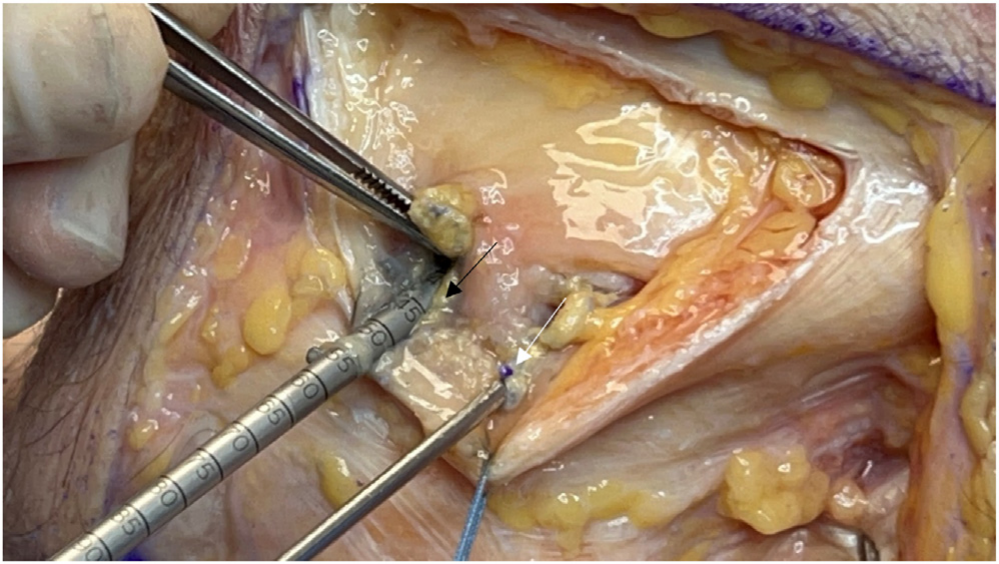
Lateral view of left knee in flexed position. A 7-mm cannulated reamer is used to over-ream the 2 guide pins to a length of 40 mm to create the fibular collateral ligament (white arrow) and popliteus (black arrow) femoral sockets.

The ITB is incised longitudinally over the lateral femoral epicondyle and elevated off the joint capsule. Traction is placed on the FCL to locate its femoral attachment. The Arthrex drill guide is positioned on the FCL attachment, aiming anteromedially, and a guide pin with a suture eyelet is drilled, exiting proximal and anterior to the adductor tubercle (Fig 10). A vertical arthrotomy is made 2 cm anterior to the FCL pin. The Arthrex Parallel Pin Guide identifies the popliteal attachment on the femur, and a second guide pin is drilled anteromedially, parallel to the FCL pin (Fig 11). Fluoroscopy is used to verify the pin positions before the pins are reamed with a 7-mm cannulated reamer to create the FCL and popliteus femoral sockets (Figs 12 and 13). Passing sutures are placed through the guide pin eyelets and pulled through the femur.

**Fig 14:**
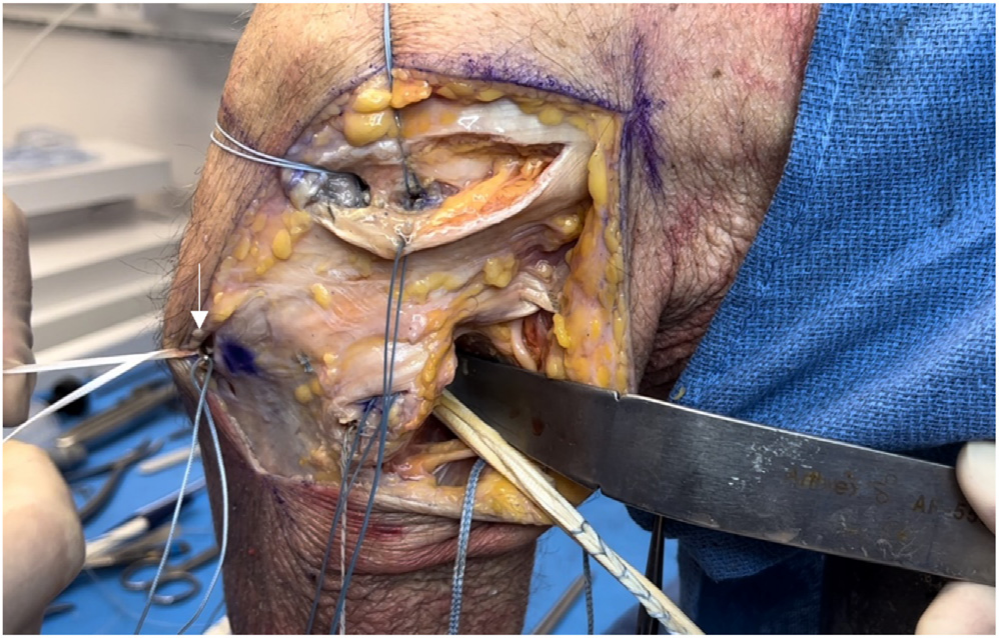
Lateral view of left knee in flexed position. By use of the tibial passing suture, the TightRope end of the graft is passed first through the tibial socket so that the button abuts the anterior tibial cortex (arrow).

**Fig 15:**
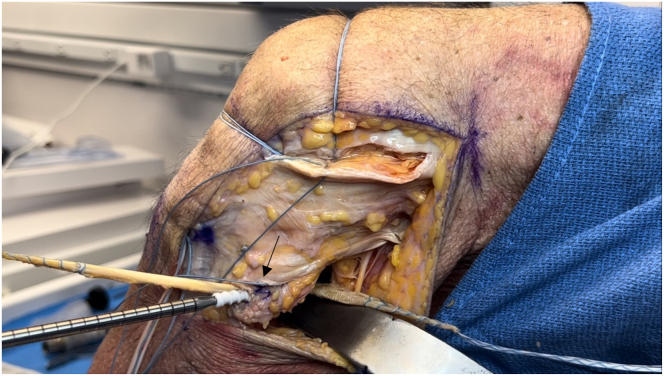
Lateral view of left knee in flexed position. The longer of the 2 graft limbs is passed posterior to anterior through the fibular tunnel (arrow) using the passing suture. The graft is then tensioned with the knee in 45° to 60° of flexion, and a 7 × 20-mm biocomposite interference screw is placed over a nitinol wire into the fibular tunnel, completing the popliteofibular ligament portion of the reconstruction.

**Fig 16:**
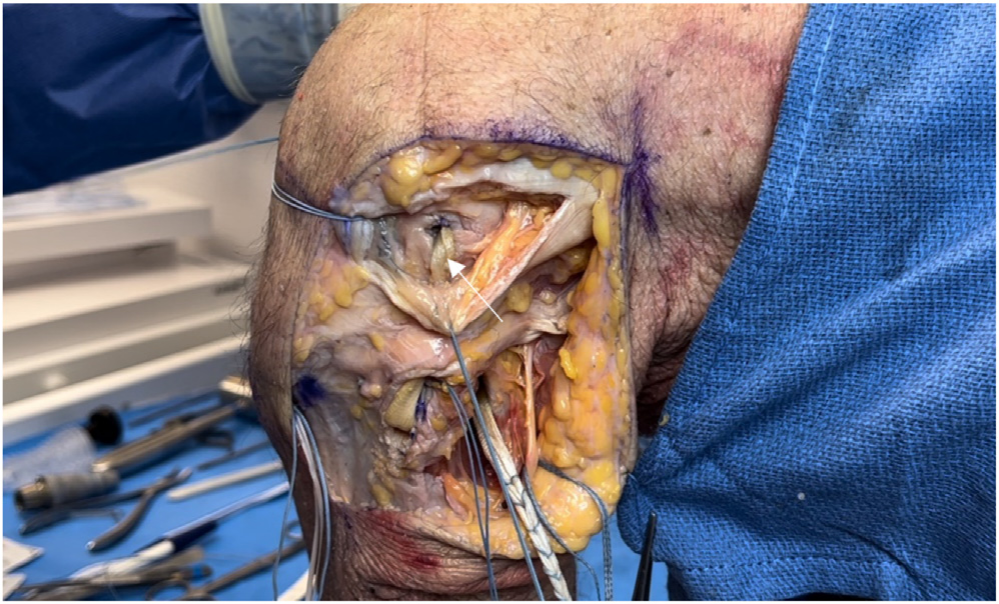
Lateral view of left knee in flexed position. The remaining graft limb that was fixed to the fibula is passed under the iliotibial band, remaining superficial to the capsule. The passing suture of the fibular collateral ligament femoral socket (arrow) is then used to shuttle the graft limb into femur.

**Fig 17:**
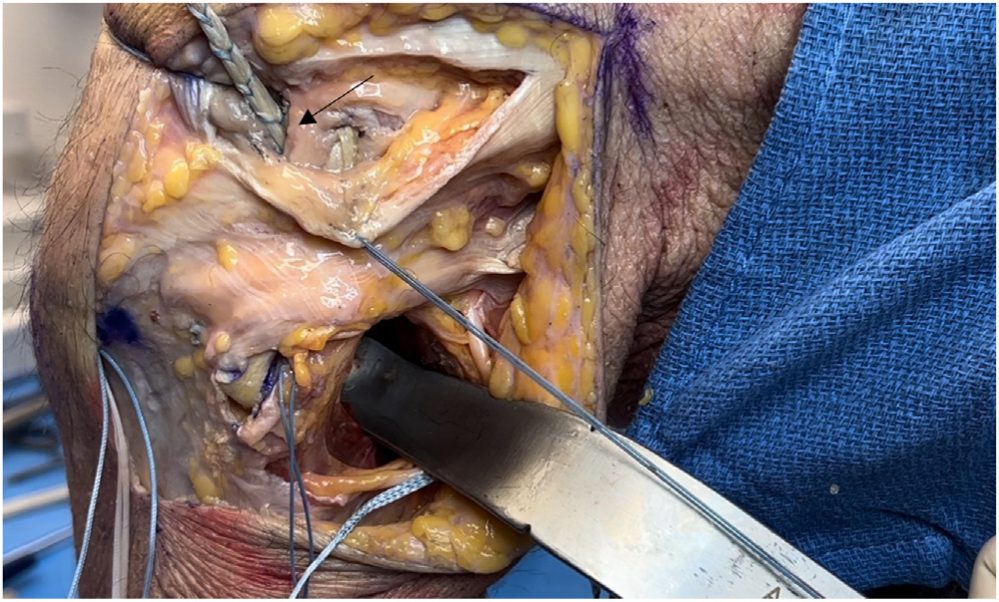
Lateral view of left knee in flexed position. The popliteal graft limb is passed deep to the joint capsule anteriorly and proximally using blunt dissection through the arthrotomy (arrow).

**Fig 18:**
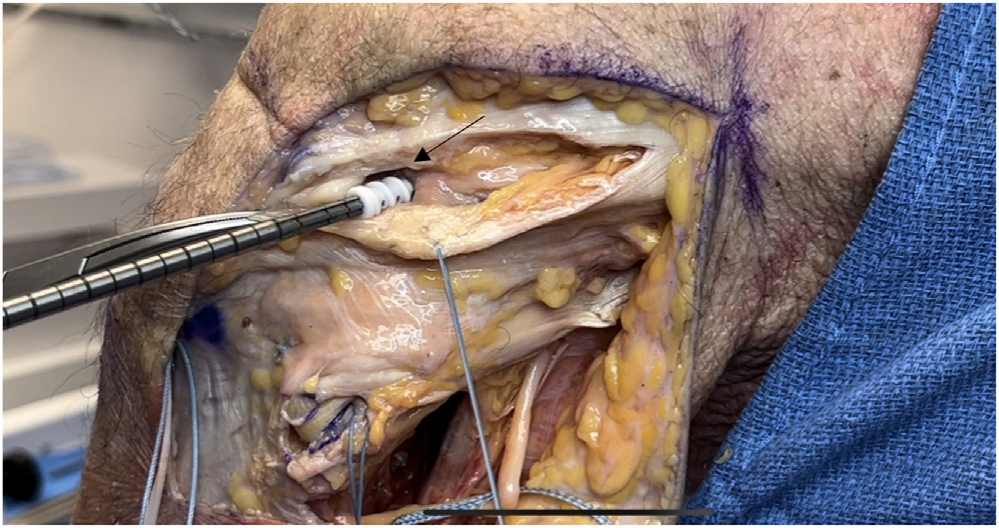
Lateral view left knee in flexed position. The popliteal graft is then passed into the popliteal femoral socket. A 7 × 20-mm biocomposite interference screw is inserted over a nitinol wire into the popliteal femoral socket (arrow) with the knee positioned in 45° to 60° of flexion, completing the popliteal portion of the reconstruction.

The graft sizing block ensures the graft passes through the tibial socket (10 mm), fibular tunnel (7 mm), and femoral sockets (7 mm). The TightRope end of the graft is passed through the tibial socket and tensioned (Fig 14). The longer graft limb is passed through the fibular tunnel, and the knee is placed in 45° to 60° of flexion with the foot neutral (Fig 15). The graft limb is tensioned, and a 6 × 20-mm or 7 × 20-mm biocomposite interference screw is inserted into the fibular tunnel. The graft limb is then passed under the ITB, and the FCL femoral socket suture shuttles the graft limb into the socket (Fig 16). The knee is positioned in 20° to 30° of flexion with a valgus force, and the graft limb is tensioned with a 7 × 20-mm screw in the femoral socket. The popliteal limb is passed deep to the joint capsule and shuttled into the femoral socket (Fig 17). The knee is placed in 45° to 60° of flexion, and a 7 × 20-mm screw is inserted into the popliteal femoral socket (Fig 18).

**Fig 19:**
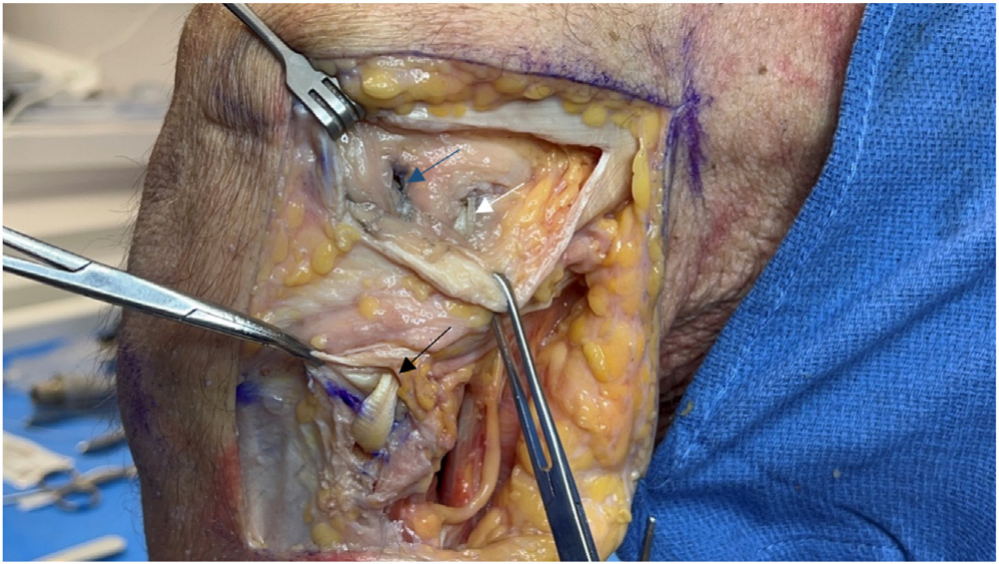
Lateral view of left knee in flexed position. Completing the reconstruction, the popliteofibular ligament (black arrow), fibular collateral ligament (white arrow), and popliteus (blue arrow) graft limbs are all examined to confirm adequate stability and fixation. A repeated examination under anesthesia with gentle varus stress is undertaken to ensure appropriate range of motion without excessive tension on the grafts.

All graft limbs and the tibial button are checked for fixation, and a repeated examination ensures appropriate motion and stability (Fig 19). The joint capsule and ITB are closed with No. 0 Vicryl sutures (Ethicon, Somerville, NJ), the biceps femoris tendon is repaired, and the subcutaneous tissues and skin are closed in layers. Pearls and pitfalls of this technique are outlined in Table 1.

**Table 1:** Surgeon Pearls and Pitfalls

Pearls
The surgeon should perform lateral dissection and tunnel drilling first (prior to addressing other intra-articular pathology, i.e., ACL and meniscal tears) for easier dissection of the common peroneal nerve and a reduced risk of potential compartment syndrome from extracapsular fluid extravasation into the thigh.
The surgeon should place a traction or tag stitch on the FCL or remnant tissue to help identify the proximal attachment site on the femur.
The surgeon should consider using intraoperative fluoroscopy to confirm adequate guide pin placement prior to drilling tunnels.
The surgeon should ensure that the femoral guide pins exit proximal and anterior to the adductor tubercle to avoid potential convergence with an ACL tunnel.
The surgeon should confirm that the graft is a minimum of 28 cm in overall length to obtain 16- and 12-cm individual graft limb lengths.
Pitfalls
Drilling a tibial socket > 2 cm should be avoided because each additional centimeter of length on the tibial socket reduces the length of the 2 individual graft limbs by 1 cm on each side.
Overly aggressive elevation of the soft tissues on the posterior aspect of the knee or mishandling of the posterior curved retractor can cause iatrogenic damage to critical neurovascular structures.
Aberrant tunnel placement can lead to fibular head fracture, nonanatomic reconstruction, or convergence with other tunnels (i.e., ACL).
Failing to rasp the tunnels adequately can significantly increase the difficulty of passing the graft limbs.
Close monitoring of tourniquet time is essential to avoid associated complications such as rhabdomyolysis or compartment syndrome.

ACL, anterior cruciate ligament; FCL, fibular collateral ligament.

### Rehabilitation

Our protocol starts with the patient weight bearing flat-footed in a hinged knee brace locked in extension when ambulating and sleeping for the first 2 weeks. Motion is allowed from 0° to 45° during this period and gradually increased. At 6 to 8 weeks, weight bearing increases by 25% weekly until full weight bearing is achieved by 8 weeks; then, the brace is removed. From 8 to 16 weeks, the patient begins advanced closed-chain strengthening and gait training exercises. Stairs and running straight ahead start at 12 weeks, and jumping exercises begin at 16 weeks. At 20 to 22 weeks, the patient progresses to sprinting, backward running, cutting/pivoting, and plyometrics. Functional sport testing is performed at 6 months, with a gradual return to sports.

## Discussion

The surgical technique presented in this article is a modification of the original anatomic PLC reconstruction described by LaPrade et al.^3^ The original technique has shown restoration of near native knee kinematics in cadaveric studies and has produced promising clinical results with respect to subjective patient-reported outcomes and objective clinical stability.^3^^,^^14^ In terms of graft choice, the original LaPrade technique used an Achilles tendon allograft harvested with a bone block that was split lengthwise to create 2 separate grafts.^3^ Modern modifications have incorporated alternate sources of tendon graft, including hamstring allograft or autograft, as well as the tibialis anterior or posterior tendons.^11^^,^^13^^,^^15^

We present a modification of the LaPrade technique using a single tibialis anterior or posterior tendon allograft that still abides by the principles of anatomic reconstruction of the FCL, popliteus tendon, and popliteofibular ligament. This single-allograft technique offers the potential advantage of efficient graft preparation without the need to prepare 2 separate grafts while avoiding the potential for donor-site morbidity of an autograft harvest. This can be particularly advantageous in the setting of a multiligament knee reconstruction in which multiple concomitant procedures may need to be performed under the same anesthetic and autograft may be needed for other portions of the procedure. Table 2 summarizes the advantages and challenges associated with our technique, all of which are important to consider when performing this technically demanding procedure.

**Table 2:** Advantages and Disadvantage of PLC Reconstruction With Single Tibialis Allograft and Suspensory Tibial Fixation

Advantages
Use of a single allograft eliminates the need for multiple grafts and any potential donor-site morbidity from autograft.
Each limb of the reconstruction (FCL, PFL, and popliteus) can be tensioned and fixated individually.
This technique adheres to the same anatomic reconstruction principles of the original LaPrade technique.
Suspensory tibial fixation allows for final retensioning to remove graft creep if desired.
Disadvantages
The procedure is technically challenging for surgeons to perform.
Allografts may not be readily available in all settings and can be costly.
Lengthy rehabilitation is often necessary—yet similar to that of other multiligament knee reconstruction protocols.
No clear guidelines have been established for several technical aspects (i.e., graft sizing, fixation options, and biological augmentation).

FCL, fibular collateral ligament; PFL, popliteofibular ligament; PLC, posterolateral corner.

In conclusion, anatomic PLC reconstruction has shown encouraging early biomechanical and clinical results. Although modifications are promising, larger clinical studies are needed to establish their relevance. This technical note presents our modified anatomic PLC reconstruction using a single tibialis anterior or posterior allograft and suspensory tibial fixation.

## Disclosures

The authors declare the following financial interests/personal relationships which may be considered as potential competing interests: N.K. reports that equipment, drugs, or supplies were provided by Arthrex and reports a consulting or advisory relationship with Arthrex. All other authors (B.C., R.P., R.M., D.W.) declare that they have no known competing financial interests or personal relationships that could have appeared to influence the work reported in this paper.

## Supplementary Data

Video 1 Key steps of posterolateral corner (PLC) reconstruction with single tibialis allograft and suspensory tibial fixation. The graft is prepared on the GraftPro preparation system with FiberLoop whipstitches placed on both ends of the graft. The graft is passed through the loop of the ACL TightRope suture card to produce 2 limbs with minimum lengths of 12 cm and 16 cm. The split is secured with 2 independent simple FiberWire stitches. A laterally based incision is made proximally along the iliotibial band (ITB) and extending distally toward the Gerdy tubercle, between the fibular head and tibial tubercle. The common peroneal nerve is carefully dissected and protected. The soleus muscle is elevated off the posteromedial aspect of the fibula toward the posterior surface of the tibia. The Arthrex drill guide for the tibial tunnel is placed halfway between the Gerdy tubercle and the tibial tubercle, aiming toward the posterior popliteal tibial sulcus. The FlipCutter drill (Arthrex) is drilled into the tibia and deployed to a diameter of 10 mm. The tibial socket is drilled in a retrograde manner to a maximum length of 20 mm. A FiberStick is used to pass a luggage-tag stitch. The fibular collateral ligament (FCL) or remnant tissue is identified and tagged. The Arthrex fibular drill guide is used to drill a guide pin through the distal attachment site of the FCL on the fibular head, aiming toward the insertion site of the popliteofibular ligament (PFL) on the posteromedial fibular styloid. The guide pin is over-reamed with a 7-mm cannulated reamer, and a passing suture is placed. The FCL attachment site on the femur is exposed. The Arthrex drill guide is placed on the FCL attachment on the femur, and a guide pin with a suture eyelet is drilled, exiting the distal femur proximal and anterior to the adductor tubercle. After an arthrotomy is made, the Parallel Pin Guide (Arthrex) is used to place a second guide pin at the location of the popliteal attachment on the femur, approximately 18.5 mm distal and anterior to the FCL. A 7-mm cannulated reamer is used to over-ream the 2 guide pins to a length of 40 mm each for the FCL and popliteus femoral sockets. Passing sutures are pulled through both tunnels. The TightRope end of the graft is passed through the tibial socket so that the button abuts the anterior tibial cortex. The longer graft limb is passed posterior to anterior through the fibular tunnel and secured with a biocomposite interference screw. This same limb of the graft is then passed under the ITB and shuttled into the FCL femoral socket. A biocomposite interference screw is placed over a nitinol wire into the femoral socket to secure the graft. By use of blunt dissection, the popliteal limb is then passed through the arthrotomy and shuttled into the popliteal socket on the femur. The graft is secured with a biocomposite interference screw over a nitinol wire. All graft limbs and the tibial button are then thoroughly examined, and a repeated examination under anesthesia confirms stability.
